# Impact of Moving Walls and Entropy Generation on Doubly Diffusive Mixed Convection of Casson Fluid in Two-Sided Driven Enclosure

**DOI:** 10.3390/e26030245

**Published:** 2024-03-10

**Authors:** Sivanandam Sivasankaran, Marimuthu Bhuvaneswari, Abdullah K. Alzahrani

**Affiliations:** 1 Mathematical Modelling and Applied Computation Research Group, Department of Mathematics, King Abdulaziz University, Jeddah 21589, Saudi Arabia; akalzahrani@kau.edu.sa; 2 Department of Mathematics, Saveetha School of Engineering, Saveetha Institute of Medical and Technical Sciences (SIMATS), Chennai 602105, India; 3 Department of Mathematics, Kongunadu Polytechnic College, D. Gudalur, Dindigul, Tamil Nadu 624620, India; msubhuvana@yahoo.com

**Keywords:** double diffusion, entropy, mixed convection, Casson fluid, two-sided driven cavity, 76A05, 76D05, 76M12, 76R05, 80A19, 80M12

## Abstract

In this study, numerical simulations are conducted with the goal of exploring the impact of the direction of the moving wall, solute and thermal transport, and entropy production on doubly diffusive convection in a chamber occupied by a Casson liquid. Wall movement has a significant impact on convective flow, which, in turn, affects the rate of mass and heat transfer; this sparked our interest in conducting further analysis. The left and right (upright) walls are preserved with constant (but different) thermal and solutal distributions, while the horizontal boundaries are impermeable to mass transfer and insulated from heat transfer. Numerical solutions are acquired using the control volume technique. Outcomes under a variety of Casson fluid parameters, including Ri, Gr, buoyancy ratio, and direction of the moving wall(s), are explored, and the influences of entropy generation are comprehensively investigated. While the flow field consists of a single cell in case I, it is dual-cellular in case III for all values of the considered parameters. Comparing the three cases, the average heat and mass transport presented lower values in case III due to the movement of an isothermal (left) wall against the buoyant force, while these values are enhanced in case I. The obtained results are expected to be useful in thermal engineering, material, food, and chemical processing applications.

## 1. Introduction

The convective movement of non-Newtonian-type fluids in various geometries is important due to their applications in daily life and scientific requirements [1,2,3,4]. A Casson fluid (CF) is a type of non-Newtonian liquid; in particular, CF presents a shear-thinning type of non-Newtonian behavior [5,6]. The thermal mixing of non-Newtonian liquids is essential in food processing and chemical processes [1,7]. Additionally, external forces, such as wall movement, dynamically influence the flow field in various fields [8,9,10,11,12]. Hence, in this study, both mechanisms are incorporated to observe the flow and the thermal and solutal fields of a Casson liquid in a closed chamber. This survey initially describes the convective flow of Casson fluids; second, single or double lid-driven cavity (LDC) flows are detailed; and finally, the entropy generation (EG) of convection flows in the enclosure is discussed. 

The convective flow of non-Newtonian liquids in closed chambers has been investigated in several studies performed throughout the past decade [13,14,15,16,17,18,19,20]. Pop and Sheremet [21] studied the radiative and buoyant convection of CF in a bounded domain with the existence of viscous dissipation. They proved that the CF parameter favors stream intensification and heat transfer augmentation. Sheremet and Pop [22] studied the (thermal) radiation and convective current of a CF in a square chamber, and achieved thermal energy drops after raising the elastic number. Hamid et al. [23] studied the free convective stream of CF in a partly heated trapezoidal chamber using the Galerkin-type finite element method (GFEM), and detected that the CF parameter decreased the velocity. Hussain et al. [24] have determined the effects of entropy generation and magnetic strength on the doubly diffusive convection of CF fluid in a staggered cavity using GFEM. The magnetic convection energy transport of CF driven by combined solutal and thermal diffusion in a trapezoidal enclosure has been explored by Shahzad et al. [25], where the impact of a tilted magnetic field ruled by the Lorentz field law force was considered. Using FEM, Aghighi et al. [26] carried out numerical research on doubly diffusive convective movement of a CF based on a viscoplastic stress model in a square-shaped chamber. They deduced that the buoyancy ratio increases the extreme yield stress by up to 400%, and that the Lewis number provides a maximum value of only 20%. Hussain et al. [27] have studied EG for the free convection of CF in a partially bottom-heated (square-shaped) porous domain with horizontal magnetic force strength, inclined angle, and viscous dissipation. GFEM was used to obtain the solution. They found that the entire entropy production rises when increasing the inclination of the domain. Hirpho and Ibrahim [28] examined the combined convection of a hybrid Casson nanofluid (CNF) in a partly heated cavity using FEM. They found that the thermal transport behavior strongly varied with an increase in the Richardson numbers. Using GFEM, Vishnu Ganesh et al. [29] investigated convective flow CNF in a complex wavy chamber with an inner solid body. Patel et al. [30] investigated thermal radiation and Dufour and Soret effects on Casson nanoliquid flow in a channel with entropy generation.

The flow due to a moving lid or wall of a cavity together with heat and solute transfer is an important problem in the area of fluid dynamics and thermal sciences, due to its extensive range of applications in metal casting, mixing, drying dynamics of lakes, cooling areas, and so on [31,32,33,34]. Some studies on a lid-driven cavity (LDC) have been performed by Sivasankaran et al. [8,9,10,11,12,31,32,33,34]; however, these studies were limited to only single-wall (top or side) motion of the cavities. Two-sided lid-driven cavity (2SLDC) flow has raised interest due to its applications in various fields, such as material processing, coating, and cooling. Some notable investigations focused on 2SLDC can be found in [35]. Multiple solutions of convective flow in a 2SLDC with an aspect ratio of 1.96 were examined by Luo and Yang [36]. Chen et al. [37] explored the effect of parallel and antiparallel motions of the (horizontal) walls of 2SLDC on steady convective flow. Asiaei et al. [38] explored the impact of multi-layered porous materials on the combined convective flow of nanoliquid in a 2SLDC, and also examined the effect of interior heat generation and EG. They found that greater thermal transport yields higher values of the entropy generation number. Maghsoudi and Siavashi [39] optimized the pore size of the porous structure in a mixed convection nanoliquid in a 2SLDC to enhance energy transport. Bhopalam et al. [40] studied the incompressible flow in an oscillating 2SLDC, and established that the dynamics of the vortex in oscillatory LDC flow is more complex than that in steady LDC flow. Azzouz and Houat [41] explored the complex topological flow pattern of a three-sided lid-driven cavity (3SLDC), showing that three types of direction support multiple alterations in the flow pattern. The LDC flow in a cavity with a top-lid, divided into two parts that could act as stirrers during a chemical mixing operation, was investigated by Turkyilmazoglu [42]. The right part moved at a uniform speed, while the left part either moved or remained stationary. Kashyap and Dass [43] examined the tilting angle effect of the 2SLDC with a hot porous block placed at the center of the cavity on the mixed convective flow under seven distinct cavity inclinations. They deduced that the titling angle of the 2SLDC has a substantial impact on thermal transport as well as the generation of entropy. Chowdhury and Rathish Kumar [44] examined the impact of the aspect ratio of the 2SLDC on the convective current of a non-Newtonian-type liquid in a 2SLDC with moving horizontal (top/bottom) boundary walls.

The amount of entropy created by any type of irreversible process—including mass and heat transfer, chemical reactions, viscous flow, and Joule heating—is known as entropy generation (EG) [45,46,47,48,49]. Every process in nature yields either zero (0) or positive values for the EG rate. This is a crucial component of the second law of thermodynamics. The analysis of EG in closed domains has been considered in several studies [45,46,47,48,49].

A considerable number of studies in the literature have focused on the convective current of non-Newtonian fluids in a closed domain. However, few studies have dealt with the convective flow of CF in a closed domain while considering the mass (solute) transfer effect. Furthermore, the influence of wall movement on Casson liquid streams has not yet been examined in the existing literature. This concept provides the main motivation for the current study: to numerically survey the interaction between wall movement and the doubly diffusive convection of CF inside a rectangular chamber, while also taking EG into consideration.

## 2. Mathematical Model 

**Figure 1:**
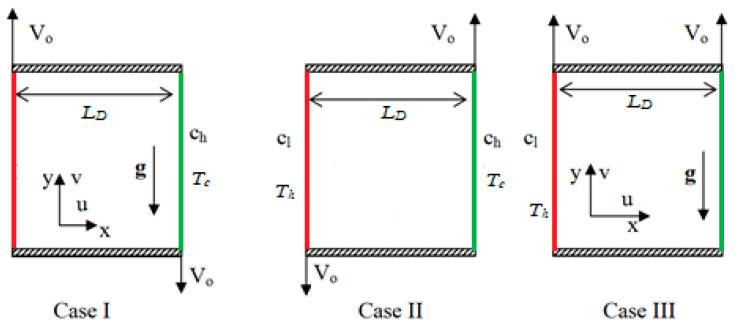
Physical configuration with three cases.

We consider the time-dependent incompressible laminar Casson liquid flow in a two-dimensional wall-driven square enclosed chamber of length L_D_, as specified in Figure 1. The thermal conditions are constant on the vertical sides and thermal insulation is executed on the horizontal sides; furthermore, constant solute conditions are applied to the vertical sides, and the mass impermeable condition is applied to the horizontal sides. The (vertical) walls of the enclosed chamber move at a constant speed (*V_o_*) in different or matching directions. Three cases were analyzed according to the movement of the wall(s). The direction of gravity’s influence is negative in the *y*-axis. The liquid’s thermal properties remain unchanged, with the exception of density (Boussinesq approximation), which is varied as follows:

(1) $$\rho =[1-\beta_{s}(c-c_{c})-\beta_{T}(T-T_{c})]\rho_{0},$$

where *β_s_* and *β_T_* are solutal and thermal expansion coefficients, respectively. It is supposed that the liquid and foreign mass do not react chemically. 

The CF rheological model is presented below [21,22]:

(2) $$\tau_{ij}=\left\{\begin{array}{c} 2\left(\mu_{pd}+\frac{P_{ys}}{\sqrt{2\pi_{C}}}\right)e_{ij},\;\;\;\;\;\;\;\;\;\;\pi <\pi_{C}\\ 2\left(\mu_{pd}+\frac{P_{ys}}{\sqrt{2\pi }}\right)e_{ij},\;\;\;\;\;\;\;\;\;\;\pi >\pi_{C} \end{array}\right.,$$

where $\pi =e_{ij}\;e_{ij}$ and $e_{ij}$ are the (*i*, *j*)th elements for the rate of deformation, $\pi_{c}$ denotes a critical rate for product of deformation rate, the plastic’s dynamic viscosity is denoted by $\mu_{pd}$, and the liquid yield stress is indicated by $P_{ys}$. The Boussinesq approximation holds for the CF. Based on the aforementioned hypotheses, the main governing model equations are

(3) $$\frac{\partial u}{\;\partial x}+\frac{\partial v}{\partial y}=0,$$

(4) $$\frac{\partial u}{\partial t}+\frac{1}{\rho_{0}}\frac{\partial p}{\partial x}+\left[u\frac{\partial u}{\partial x}+v\frac{\partial u}{\partial y}\right]=\nu \left(1+\frac{1}{\beta }\right)\left[\frac{\partial^{2}u}{\partial x^{2}}+\frac{\partial^{2}u}{\partial y^{2}}\right]\;,$$

(5) $$\frac{\partial v}{\partial t}+\frac{1}{\rho_{0}}\frac{\partial p}{\partial y}+\left[u\frac{\partial v}{\partial x}+v\frac{\partial v}{\partial y}\right]=\nu \left(1+\frac{1}{\beta }\right)\left[\frac{\partial^{2}v}{\partial x^{2}}+\frac{\partial^{2}v}{\partial y^{2}}\right]+g\left[\beta_{T}(T-T_{c})+\beta_{s}(c-c_{l})\right],$$

(6) $$\frac{\partial T}{\partial t}+u\frac{\partial T}{\partial x}+v\frac{\partial T}{\partial y}-\frac{k}{\rho_{0}c_{p}}\left[\frac{\partial^{2}T}{\partial x^{2}}+\frac{\partial^{2}T}{\partial y^{2}}\right]=0,$$

(7) $$\frac{\partial c}{\partial t}+u\frac{\partial c}{\partial x}+v\frac{\partial c}{\partial y}-D\left[\frac{\partial^{2}c}{\partial x^{2}}+\frac{\partial^{2}c}{\partial y^{2}}\right]=0,$$

where β is the CF parameter. The relevant boundary conditions and initial values for the present problem are as follows:

(8) $$\begin{array}{cc} t=0:\; & v=0;\;u=0,\;\;\;T\;=T_{c\;},c=c_{l},\;0\leq (x,y)\leq L_{D},\\ t>0:\; & u=0;\;v=\pm V_{0},\;T\;=T_{h\;},\;c=c_{l},\;x=0,\\ & u=0;\;v=\pm V_{0},\;T\;=T_{c\;},\;c=c_{h},\;x=L_{D},\\ & v=0;\;u=0,\;\;\;T_{y}=c_{y}=0,\;y=L_{D}\&0. \end{array}$$

The following variables are used to perform non-dimensionalization of the governing model equations:

(9) $$(X,Y)=\frac{\left(x,y\right)}{L_{D}},\;(V,U)=\frac{\left(v,u\right)}{V_{0}},\;\theta =\frac{T-T_{c}}{\left(T_{h}-T_{c}\right)},\;C=\frac{c-c_{l}}{\left(c_{h}-c_{l}\right)},\;t*=\frac{tV_{0}}{L_{D}},\;\mathrm{and}\;P=\frac{p}{\rho_{0}{V_{0}}^{2}}.$$

The dimensionless modeled equations are as follows:

(10) $$\frac{\partial U}{\partial X}+\frac{\partial V}{\partial Y}=0$$

(11) ∂U∂t*+∂P∂X+U∂U∂X+V∂U∂Y=1Re 1β+1∇2U

(12) ∂V∂t*+∂P∂Y+U∂V∂X+V∂V∂Y=1Re 1β+1∇2V+Ri (θ+NC)

(13) $$\frac{\partial \theta }{\partial t*}+U\frac{\partial \theta }{\partial X}+V\frac{\partial \theta }{\partial Y}-\frac{1}{Re\;Pr}\left[\frac{\partial^{2}\theta }{\partial X^{2}}+\frac{\partial^{2}\theta }{\partial Y^{2}}\right]=0$$

(14) $$\frac{\partial C}{\partial t*}+U\frac{\partial C}{\partial X}+V\frac{\partial C}{\partial Y}-\frac{1}{Re\;Sc}\left[\frac{\partial^{2}C}{\partial X^{2}}+\frac{\partial^{2}C}{\partial Y^{2}}\right]=0,$$

where N=GrsGrT is the buoyancy ratio, ${Gr}_{T}=\frac{\beta_{T}g\Delta T{L_{D}}^{3}}{{\left(\nu \right)}^{2}}$ is the thermal Grashof number, ${Gr}_{s}=\frac{\beta_{s}g\Delta C{L_{D}}^{3}}{{\left(\nu \right)}^{2}}$ is the solutal Grashof number, $Re=\frac{V_{0}L_{D}}{\nu }$ is the Reynolds number, $Pr=\frac{\nu }{\alpha }(=10)$ is the Prandtl number, Ri=GrTRe2 is the Richardson number, and the Schmidt number is $Sc=\frac{\nu }{D}$. 

The initial and boundary values, expressed in dimensionless form, are as follows:

(15) $$\begin{array}{cc} t*=0: & \;\;V=U=0,\;C=\theta =0,\;\;\;0\leq Y,X\leq 1,\\ t*>0: & \;\;V=U=0,\;C_{Y}=\theta_{Y}=0,\;Y=1\&0,\\ & \;\;V=\pm 1;\;U=0;\;\;\theta =1;\;C=0;\;X=0,\\ & \;\;\;V=\pm 1;\;U=0;\;\;\theta =0;\;C=1;\;X=1. \end{array}$$

The rate of heat transport across the chamber is a crucial constraint in thermal science applications. At the left (hot) barrier, the local Nusselt number is deduced as $Nu={\left(-\frac{\partial \theta }{\partial X}\right)}_{X\;=0}$. The averaged Nusselt number, which may be stated as follows, is used to calculate the total heat transport across the chamber:

$$\;\bar{Nu}\;\;=\int_{0}^{1}NudY$$

Additionally, the local Sherwood number alongside the cold (right) barrier of the domain is derived as $Sh={\left.-\frac{\partial C}{\partial X}\right|}_{X=1}$. The whole solute transfer across the chamber is represented by the averaged Sherwood number, which is expressed as follows:

$$\;\bar{Sh}\;\;=\int_{0}^{1}ShdY$$

## 3. Analysis of Entropy Generation 

In thermal and industrial applications, buoyancy-induced convection in an enclosed chamber is of great importance. Nonetheless, the concept of entropy generation allows one to identify ideal parameters for real-world use. Entropy production may be computed from the previously known temperature, velocity, and solute concentration fields, as it is associated with the irreversible nature of heat and mass transport as well as viscosity effects. These methods can be used to calculate the (local) entropy generation per unit area of an incompressible fluid. The generation of local entropy is a result of liquid friction, heat, and mass transfer.

(16) $$S_{heat}=\frac{k}{T_{c}^{2}}\left[{\left(\frac{\partial T}{\partial x}\right)}^{2}+{\left(\frac{\partial T}{\partial y}\right)}^{2}\right]$$

(17) $$S_{con}=\frac{R_{D}}{c_{0}}\left[{\left(\frac{\partial c}{\partial x}\right)}^{2}+{\left(\frac{\partial c}{\partial y}\right)}^{2}\right]+\frac{R_{D}}{c_{0}}\left[\frac{\partial c}{\partial x}\frac{\partial T}{\partial x}+\frac{\partial c}{\partial y}\frac{\partial T}{\partial y}\right]$$

(18) Sfluid=(1+1β)  μ Tc2∂u∂x2+∂v∂y2+∂u∂y+∂v∂x2.

The three quantities mentioned above are added to obtain the total (local) entropy production:

(19) SGen=kTc2∂T∂x2+∂T∂y2+RDc0∂c∂x2+∂c∂y2+RDc0∂c∂x∂T∂x+∂c∂y∂T∂y+(1+1β)  μ Tc2∂u∂x2+∂v∂y2+∂u∂y+∂v∂x2.

Applying (10) in a conventional manner, the non-dimensional formula for entropy production is achieved.

(20) $$S_{heat}^{*}={\left(\frac{\partial \theta }{\partial X}\right)}^{2}+{\left(\frac{\partial \theta }{\partial Y}\right)}^{2}$$

(21) $$S_{con}^{*}=\phi_{c1}\left[{\left(\frac{\partial C}{\partial X}\right)}^{2}+{\left(\frac{\partial C}{\partial Y}\right)}^{2}\right]+\phi_{c2}\left[\frac{\partial C}{\partial X}\frac{\partial \theta }{\partial X}+\frac{\partial C}{\partial Y}\frac{\partial \theta }{\partial Y}\right]$$

(22) $$S_{fluid}^{*}=\phi_{f1}\left\{{\left(\frac{\partial U}{\partial Y}+\frac{\partial V}{\partial X}\right)}^{2}+2\left[{\left(\frac{\partial U}{\partial X}\right)}^{2}+{\left(\frac{\partial V}{\partial Y}\right)}^{2}\right]\right\}\left(\frac{1}{\beta }+1\right)$$

$$S_{total}=S_{heat}^{*}+{S_{con}^{*}+S}_{fluid}^{*}.$$

Integrating the (dimensionless) local entropy throughout the system, one can obtain the total entropy production:

$${SG}_{total}=\int_{V}S_{total}\left(X,Y\right)dA.$$

The ratio of total irreversibility to heat transmission irreversibility is described by the local Bejan number:

$${Be}_{loc}=\frac{S_{heat}^{*}}{S_{total}}.$$

When ${Be}_{loc}>\frac{1}{2}$, the irreversibility of heat transmission predominates; when ${Be}_{loc}<\frac{1}{2}$, the irreversibility of fluid resistance predominates. Heat transport and viscous irreversibilities are equivalent at ${Be}_{loc}=\frac{1}{2}$. The average Bejan number is employed in this study to establish the relative significance of the irreversibility of heat transmission across the domain of interest.

$$Be=\frac{\int_{A}{Be}_{loc}\left(X,Y\right)dA}{\int_{A}dA}.$$

The ForTran-90 computer software, which was developed for temperature and stream fields, was expanded to compute the formation of entropy within the chamber.

## 4. Numerical Technique and Validation 

As the FVM is a very famous technique for solving fluid flow problems, it was used to solve the governing model (non-dimensional) Equations (10)–(14), subject to the boundary constraints (15). Convective and diffusive terms were discretized using the QUICK and central difference techniques, respectively. A set number of non-overlapping control volumes were used to sub-divide the computational domain of interest. A test for grid independency was executed with different grid sizes from 42 × 42 to 162 × 162. It was found that a grid of 122 × 122 provided a grid-independent solution. The tri-diagonal matrix method was used to carry out the iterative procedure. The iteration’s convergence condition, which is 10^−6^, was maintained. Validation of the present computational code was conducted against existing outcomes for natural convection in a closed cavity [50], as detailed in Table 1. Consistency across the outcomes ensured that the current code for studying the topic is numerically accurate. All numerical simulations were performed using ForTran 90 in-house code.

## 5. Results and Discussion

The purpose of the conducted numerical study was to determine how moving-wall orientation, heat, and mass transfer affect the doubly diffusive convection current in a square box occupied with CF. The CF parameter, moving wall direction, solutal Grashof number ($Gr_{s}=10^{4}\&10^{6}$), thermal Grashof number ($Gr_{T}=10^{4}\&10^{6}$), Reynolds number (Re=10 to 103), Richardson number (0.01≤Ri≤100), and CF parameter (0.01≤β≤1) were the governing parameters in the experiment. To examine the different effects, simulations were performed under a variety of combinations of the aforementioned parameters. The influence of the moving direction of the vertical isothermal walls was explored in mixed convective flow with heat and mass distribution of Casson liquids in a container. Three cases were considered, which were designed according to different directions of movement; namely, in case I, the hot (left-side) wall moves up while the cold wall (right-side) moves down, while the hot wall moves down while the cold wall moves up in case II. Both walls move up in case III, as shown in Figure 1.

**Figure 2:**
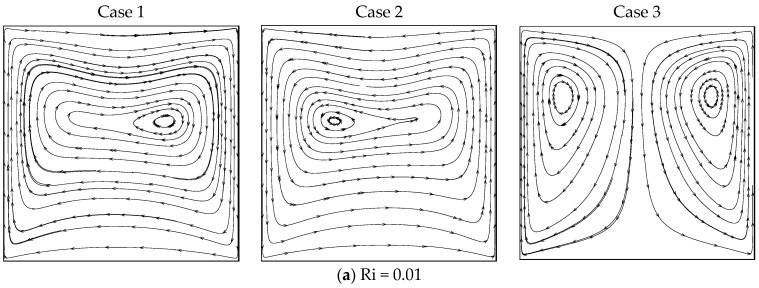

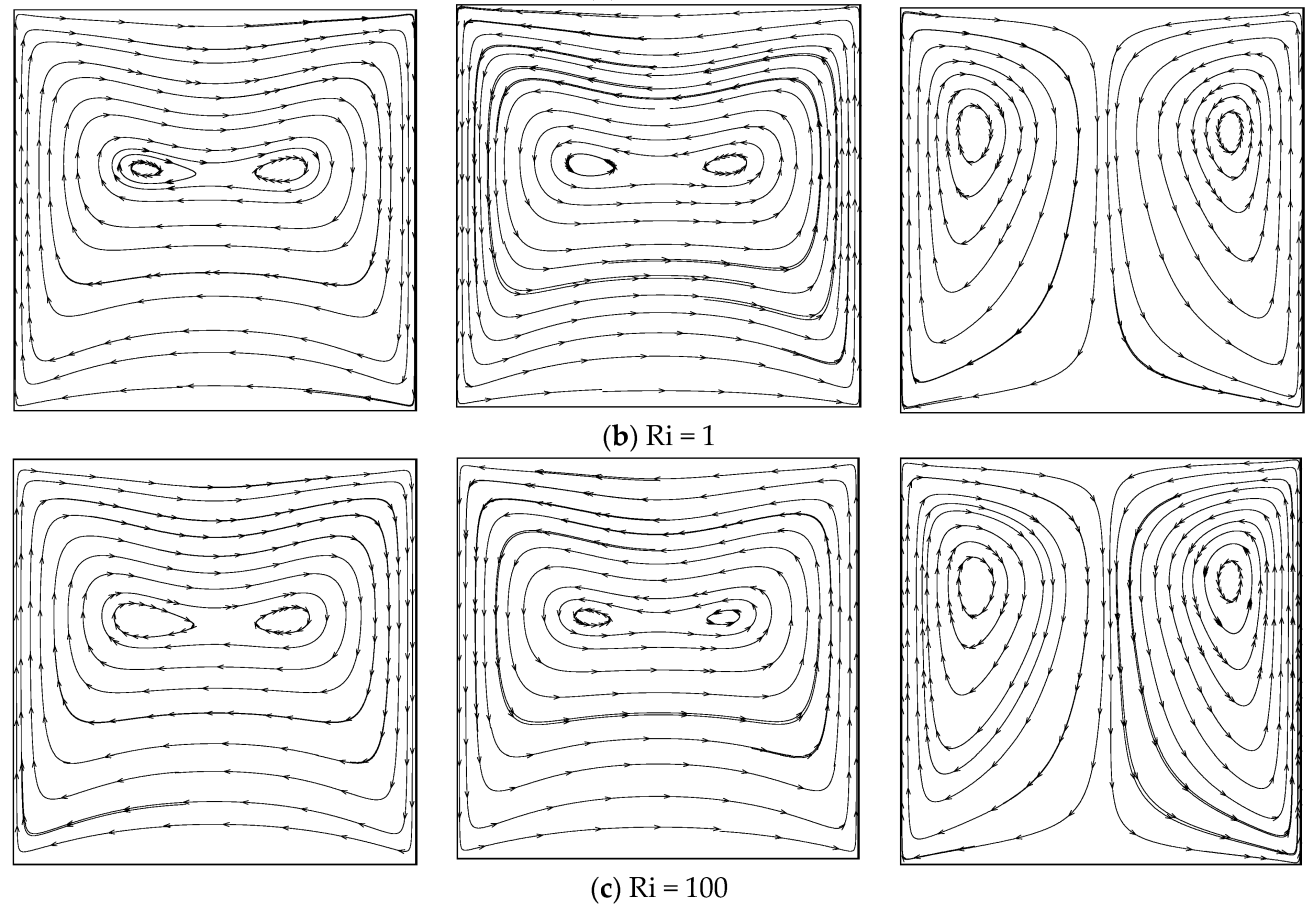
Streamlines for different Ri under the three cases with β = 0.01.

Figure 2 shows the streamlines obtained with different values of *Ri* under the three cases of wall movement with β = 0.01. There is just one clockwise rotating eddy for all values of *Ri* in case I, where one eddy fills the entire domain as a result of the buoyancy force and the heated barrier moving upward. The eddy’s core region elongates horizontally, and two inner eddies form when *Ri* = 1 and *Ri* = 100. The opposite trend was observed in case II; that is, a single anti-clockwise rotating eddy was observed inside the cavity. Here, the hot (left) wall moves down and against the buoyancy force near the isothermal wall. The cell stretched horizontally for all Ri numbers in both cases I and II. However, the stream pattern was totally different in case III, where both barriers moved upwards. This resulted in a dual cell structure for all Ri values. When both walls moved in the same direction (i.e., upwards), the shear force drove the liquid near the wall to move up and form a dual cell pattern. The mechanism of heat transmission changed as the Richardson number increased, but the flow structure was not significantly impacted.

**Figure 3:**
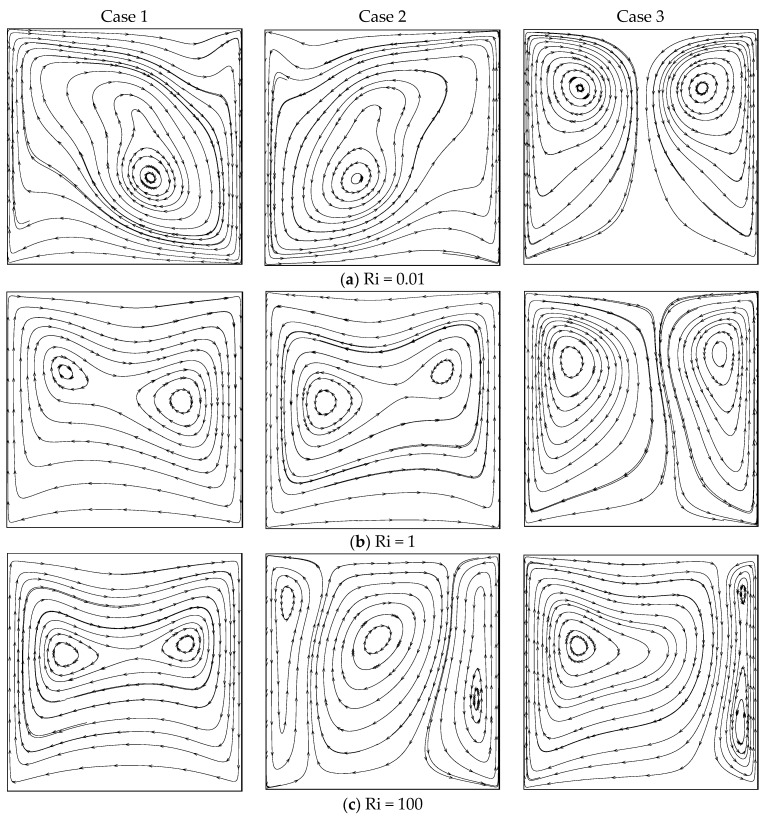
Streamlines for different Ri values under the three cases with β = 1.

Figure 3 displays the flow patterns under the three cases and three modes of heat transfer with β = 1. Although a single cell pattern was observed for all Ri values in case I, the location of the eddy core region depended on the value of Ri. In the forced convection regime (Ri=0.01), the cell stretched diagonally and the core region of the eddy was observed at the lower left portion of the cavity. When Ri = 1 and 100 (i.e., in mixed and natural convection regimes), the cell stretched horizontally and two inner cells formed in the core area of the eddy. When considering case II, an anti-clockwise rotating single cell formed under forced and mixed convection regimes. Nevertheless, the flow pattern completely changed in the free convection regime, where a tri-cellular pattern was observed. This flow structure was not observed for lower values of β. The dual eddy arrangement formed for all values of Ri in case III. However, the size of the cells depended on the value of *R;* that is, the mode of heat transfer. In the forced convective flow regime, the two eddies were of the same size and power, and the cell generated by the hot wall became strengthened. Further increasing *Ri* (Ri=100), the hot cell gained more strength and occupied the majority of the container. Consequently, the eddy generated by the cold wall shrank in size. Comparing Figure 2 and Figure 3, the stream pattern was affected much more in the case β = 1, compared to that under β = 0.01, when changing the values of Ri.

**Figure 4:**
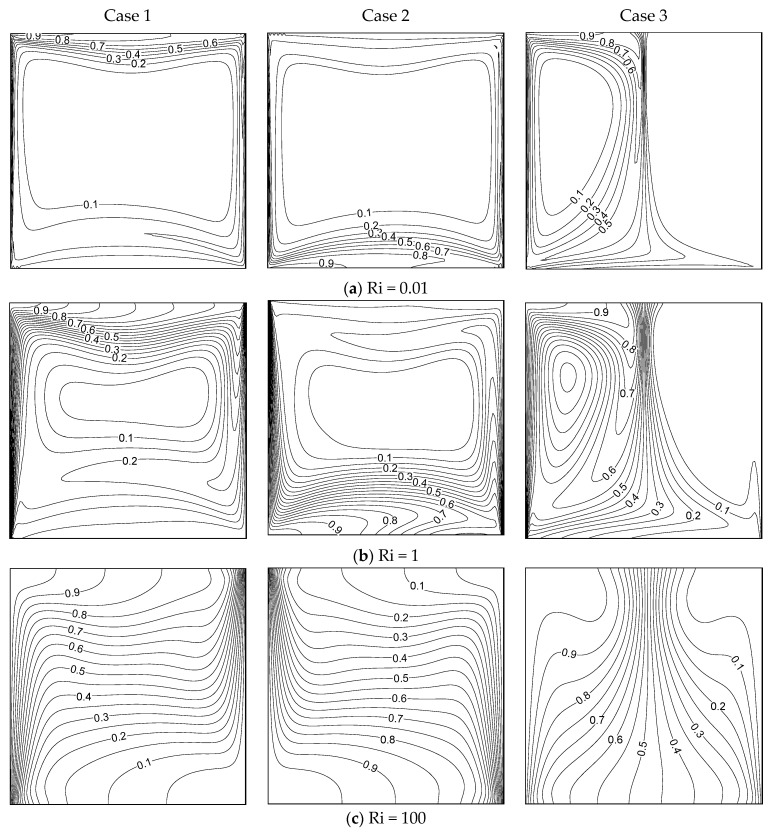
Isotherms for different Ri values and the three cases with β = 0.01.

**Figure 5:**
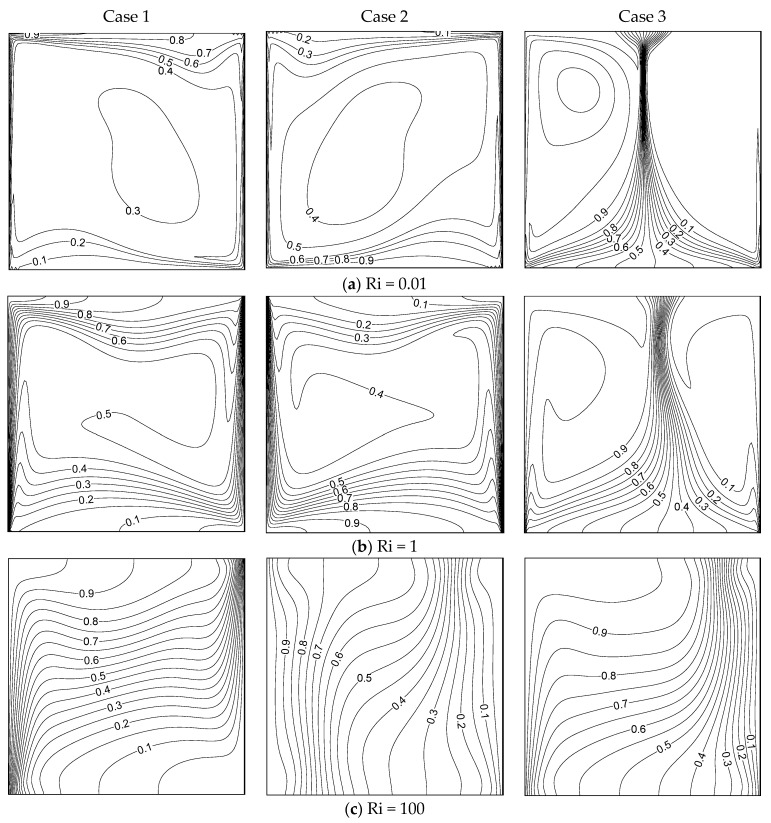
Isotherms for different Ri values under the three cases with β = 1.

Figure 4 demonstrates the thermal distribution inside the container for different Ri and wall movement directions (three cases) with β = 0.01. In case I, thermal boundary layers formed along the moving isothermal walls. Higher thermal gradients were found at the top portion of the container under Ri = 0.01. In forced convection (Ri = 100), the isotherms were distributed equally inside the container, and vertical thermal stratification was observed. However, higher thermal gradients were found at the bottom of the cavity for Ri = 0.01 and 1 in case II. When the walls moved in the same direction (case III), the thermal distribution totally varied from case I and case II. At Ri = 0.01, a high thermal gradient existed at the central part of the container for Ri=0.01 and1. When Ri = 100, the isotherms were distributed over almost the whole container. The convection mode of heat transmission was predominant in every case examined here. Figure 5 displays the thermal pattern inside the container for the three cases of moving walls and under different values of Ri with β = 1. Every figure demonstrates the convection mechanism of heat transmission in detail. Strong thermal boundary layers developed alongside the isothermal walls at Ri=0.01 and 1 in cases I and II. In the free convection regime, the isotherms dispersed effectively within the container. No thermal boundary layer existed in case III. However, there were significant temperature variations in the cavity’s center (X=0.5). When increasing Ri=1, the thermal gradient moved slightly towards the right side. At Ri = 100, these thermal gradients appeared close to the cold wall.

**Figure 6:**
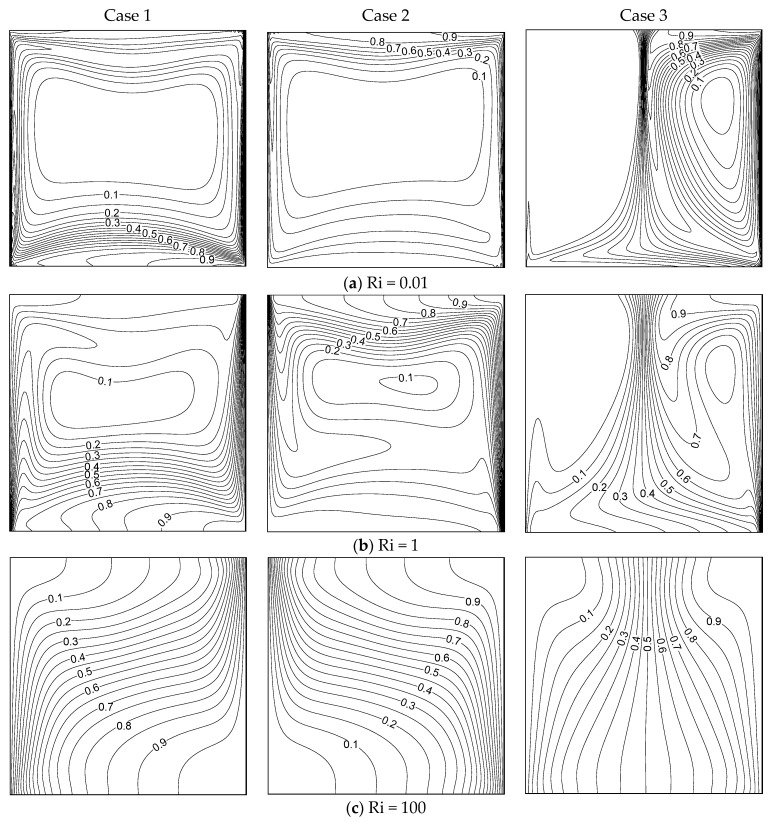
Isomasslines for different Ri values under the three cases with β = 0.01.

**Figure 7:**
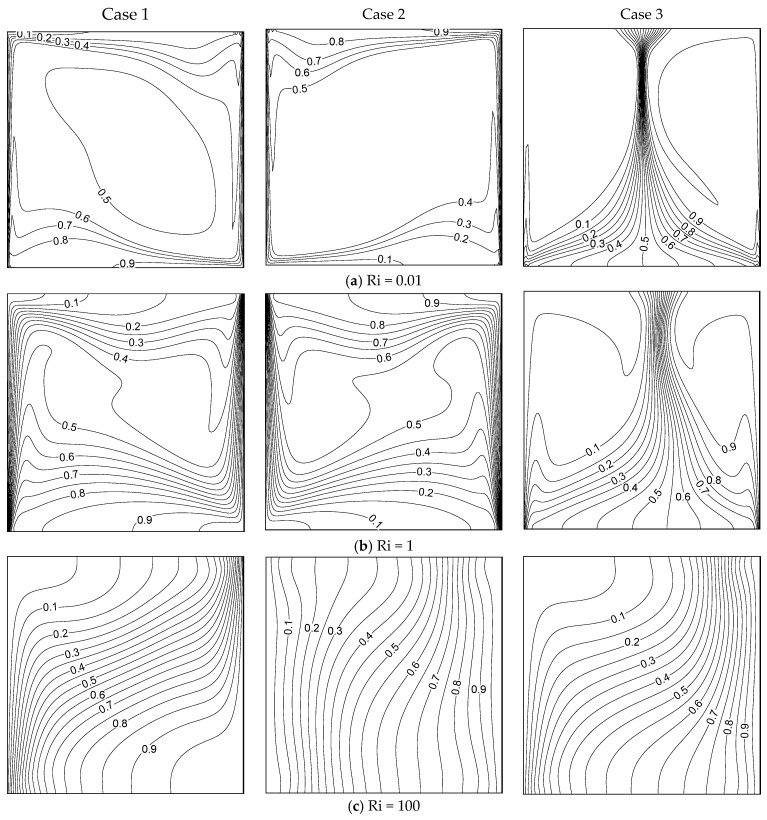
Isomasslines for different Ri values under the three cases with β = 1.

Figure 6 demonstrates the solute distributions inside the container for diverse values of Ri and the three cases of moving wall directions with β = 0.01. The solutal mixing inside the container was strong in case I, and solutal gradient layers formed along the moving vertical barriers at Ri=0.01and 1 for cases I and II. We observed a similar trend of solutal mixing while both walls were moving oppositely (Cases I and II). As the walls in cases I and II were moving in the same direction, the solute mixing in case III completely differed from that in cases I and II. Here, a very strong solutal gradient existed in the middle of the container, due to the dual cell stream pattern due to moving the wall in the same direction. The isomasslines were distributed equally inside the container at Ri = 100 for all cases of the moving walls. Figure 7 demonstrates the solutal mixing inside the container for various values of Ri and the three cases of moving walls with β = 1. It can be seen, from these graphs, that the mass transmission is clearly influenced by the wall’s movement and its direction. The liquid was well-mixed inside the container in all cases, boosting the strong solutal mixing. The mass distribution showed a similar trend as discussed for Figure 6 in the considered cases. Comparing Figure 6 and Figure 7, the influence of the CF parameter on mass transfer can be clearly understood. 

**Figure 8:**
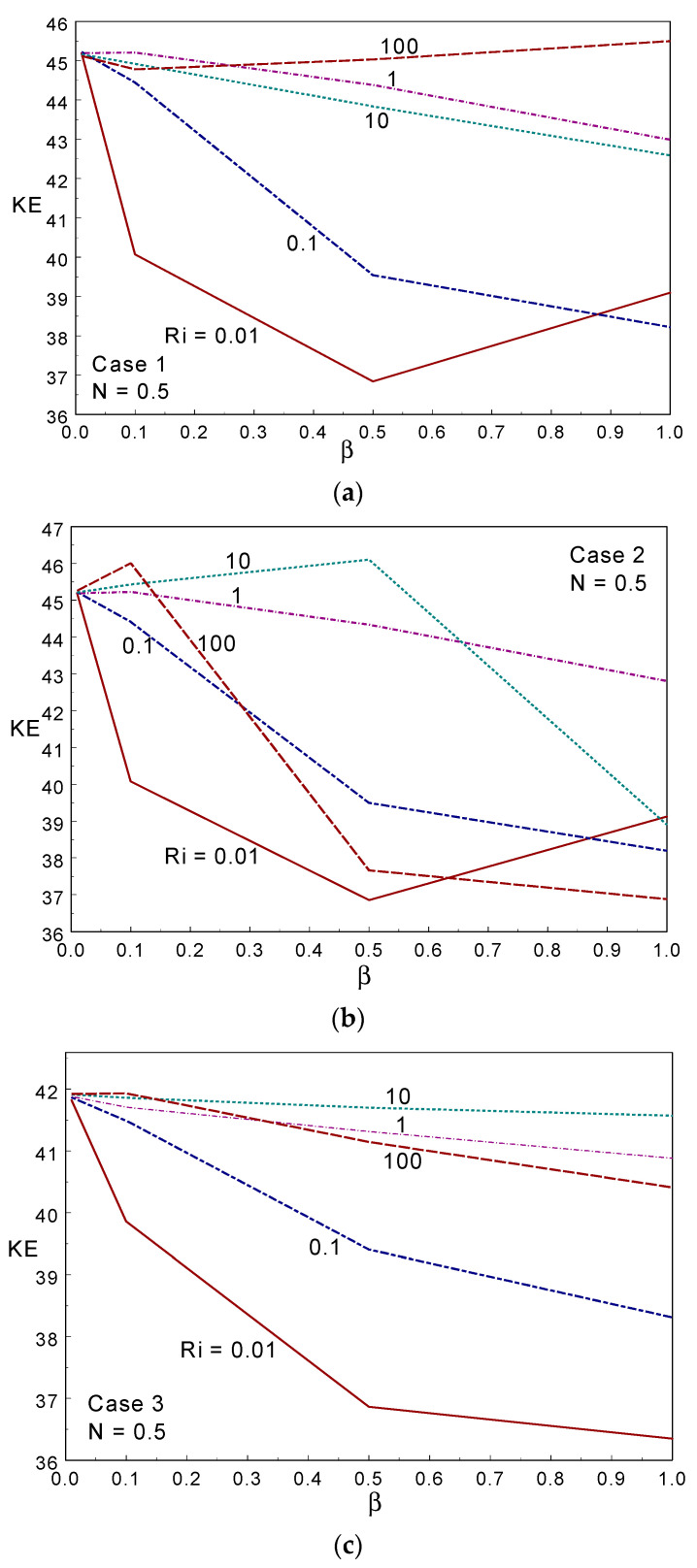
Kinetic energy for different values of the Casson fluid parameter and Ri in Case 1 (**a**), Case 2 (**b**), and Case 3 (**c**).

**Figure 9:**
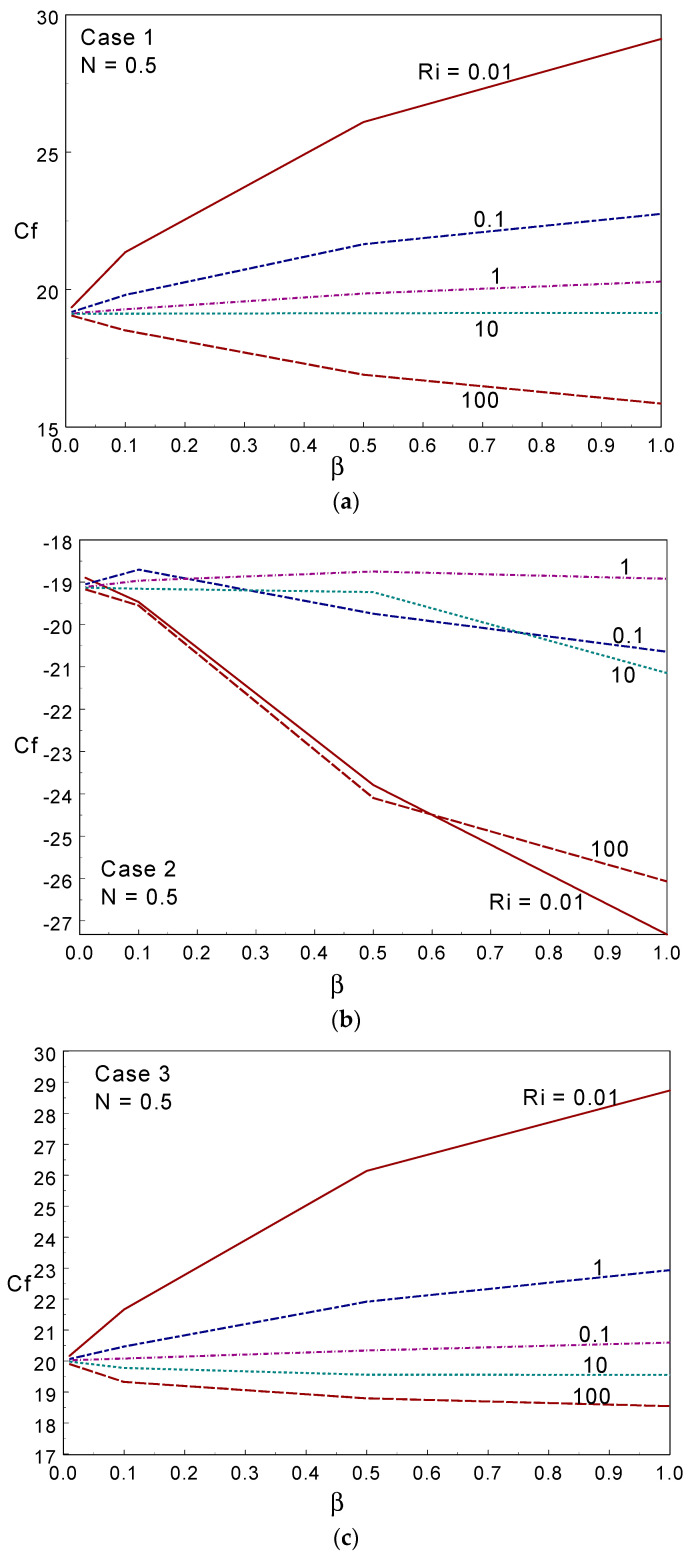
Skin friction for different values of the Casson fluid parameter and Ri in Case 1 (**a**), Case 2 (**b**), and Case 3 (**c**).

The kinetic energy (KE) obtained under different values of the Casson fluid parameter, Ri, and the three cases are displayed in Figure 8. The KE behaved non-linearly with increasing values of Ri, and declined in most of the considered cases when increasing the Casson fluid parameter. Case III provided a lower KE than the other two cases in most of the simulations. Figure 9 shows the skin friction (SF) along the moving right wall under numerous values of β, Ri, and the three cases of the moving wall directions with N = 0.5. The skin friction increased when increasing the value of β at Ri≤1, was almost constant when Ri = 10, and declined when Ri = 100 in case I. As the hot (left) wall moves up in cases I and III, a similar trend of variation in SF was observed when changing the *Ri* and β values. The SF declined when increasing the Ri values for all β in cases I and III. The deviation of the SF value between the two Ris increased when intensifying the CF parameter; that is, the SF increased (decreased) when increasing the value of the CF parameter under forced and mixed (free) convection regimes. When considering case II, SF declined when increasing the β value; in particular, the skin friction declined with increasing β for all Ri except Ri = 1 in case II. When Ri = 1, SF behaved constantly for all values of β (Case II). Comparing the three cases, cases I and III presented higher drag along the moving wall. As the wall moved downwards and opposite to the buoyancy force in Case II, the skin friction value took a negative sign, and was lower than in the other two cases.

**Figure 10:**
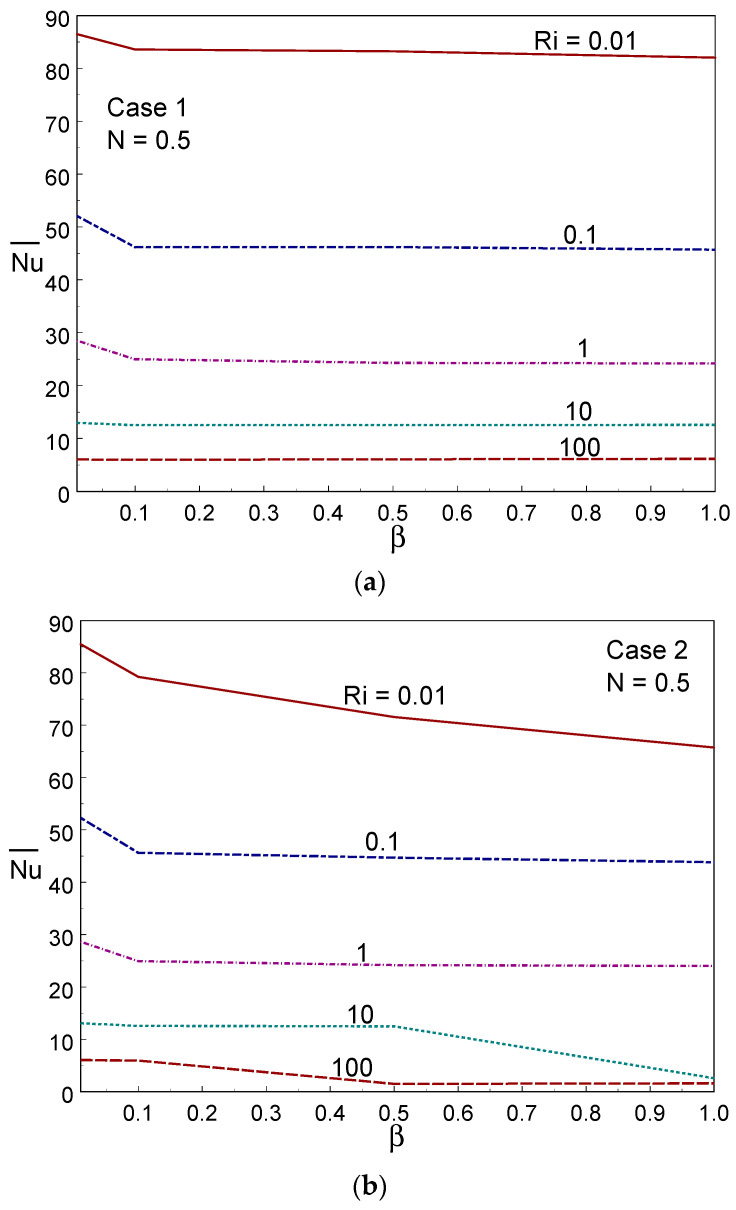

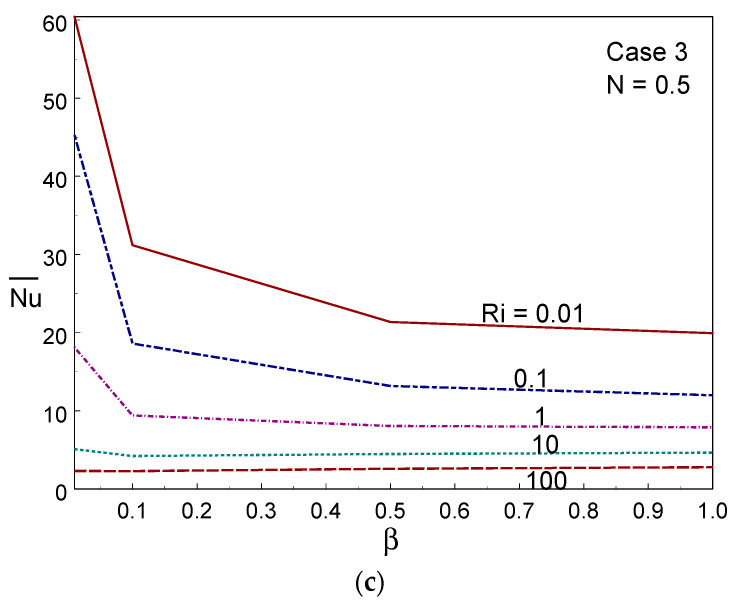
Averaged Nusselt number for different values of Ri and Casson fluid parameters in Case 1 (**a**), Case 2 (**b**), and Case 3 (**c**).

To illustrate the impact of the averaged heat transmission across the container, the averaged Nusselt number is displayed, against different CF parameter and Ri values, for the three cases in Figure 10. The averaged heat transfer rate remained constant with a change in the Casson fluid parameter when Ri≥10 in case I. When Ri≤1, the averaged heat transfer rate declined slightly first, then became constant in case I. The averaged Nusselt number declined when increasing the CF parameter in case II for all values of Ri; in particular, the averaged heat transfer experienced a substantial reduction when β changed from 0.01 to 0.1. In case III, a similar decline also occurred when β increased from 0.01 to 0.1. From the three cases, it was found that the averaged Nusselt number deteriorated when increasing the value of Ri; that is, the mode of forced convection (Ri<<1) provides higher heat transport than the free and mixed convection regimes (Ri≥1). Comparing the three cases, the averaged Nusselt number obtained lower values in case III; that is, the averaged heat transport was low in this case. This is due to the movement of an isothermal (left-side) wall against the buoyancy force. However, the left hot wall moves up, supporting the buoyancy force to produce a higher heat transmission rate in cases I and III. The natural convection mode was not influenced significantly by the heat transmission rate when varying the CF parameter values in all three cases. 

**Figure 11:**
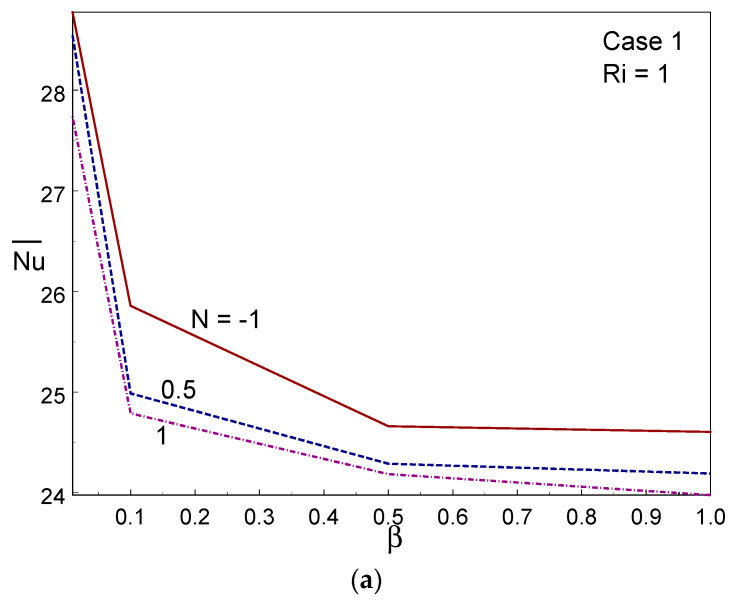

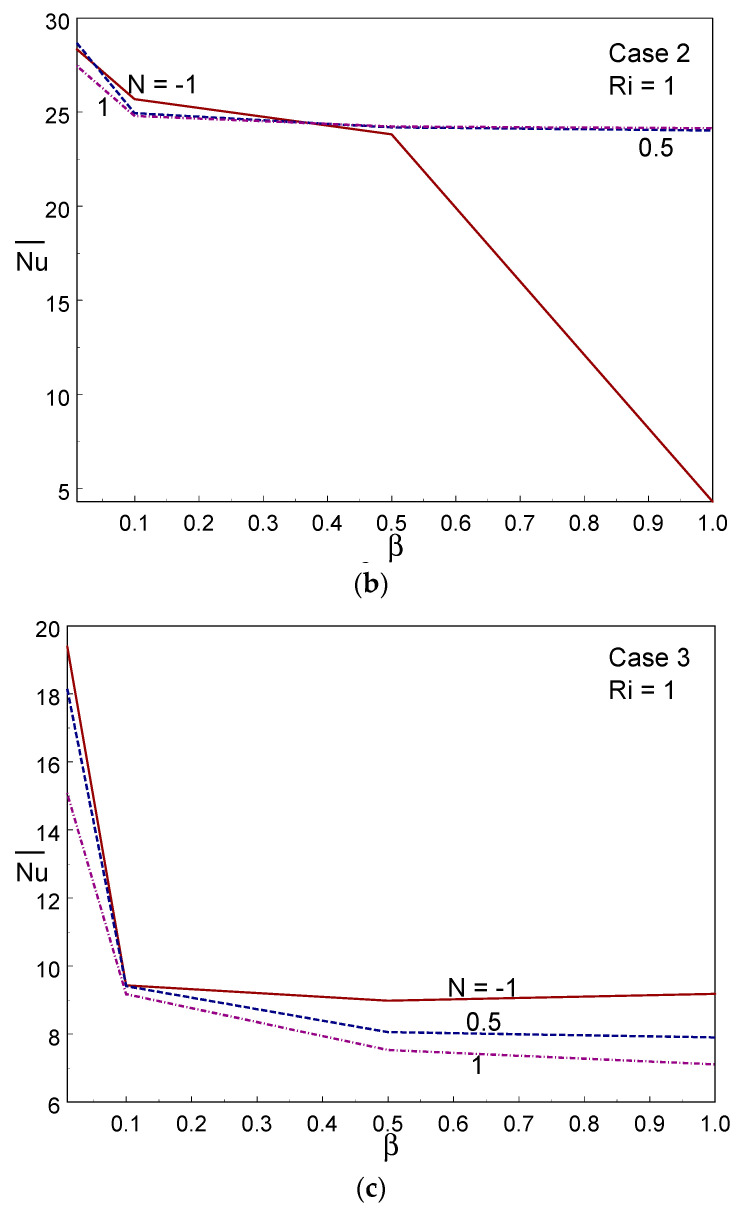
Averaged Nusselt number for different values of N and Casson fluid parameters in Case 1 (**a**), Case 2 (**b**), and Case 3 (**c**).

Figure 11 displays the averaged thermal transfer rate across the container against the CF parameter and buoyancy ratio parameter (N) for the three cases. It can be observed, from Figure 11, that case I offered the higher heat transport rate among the three cases, whereas case III provided a lower heat transport rate. When raising the buoyancy ratio, the averaged Nusselt number declined in cases I and III, while the opposite trend was observed in case II. When changing the Casson liquid parameter from 0.01 to 0.1, a sharp decrease in the averaged Nusselt number was found in Cases I and II, after which the averaged Nusselt number declined gradually with increasing β. The average heat transmission decreased as the CF parameter increased, as this results in higher friction between the liquid particles. 

**Figure 12:**
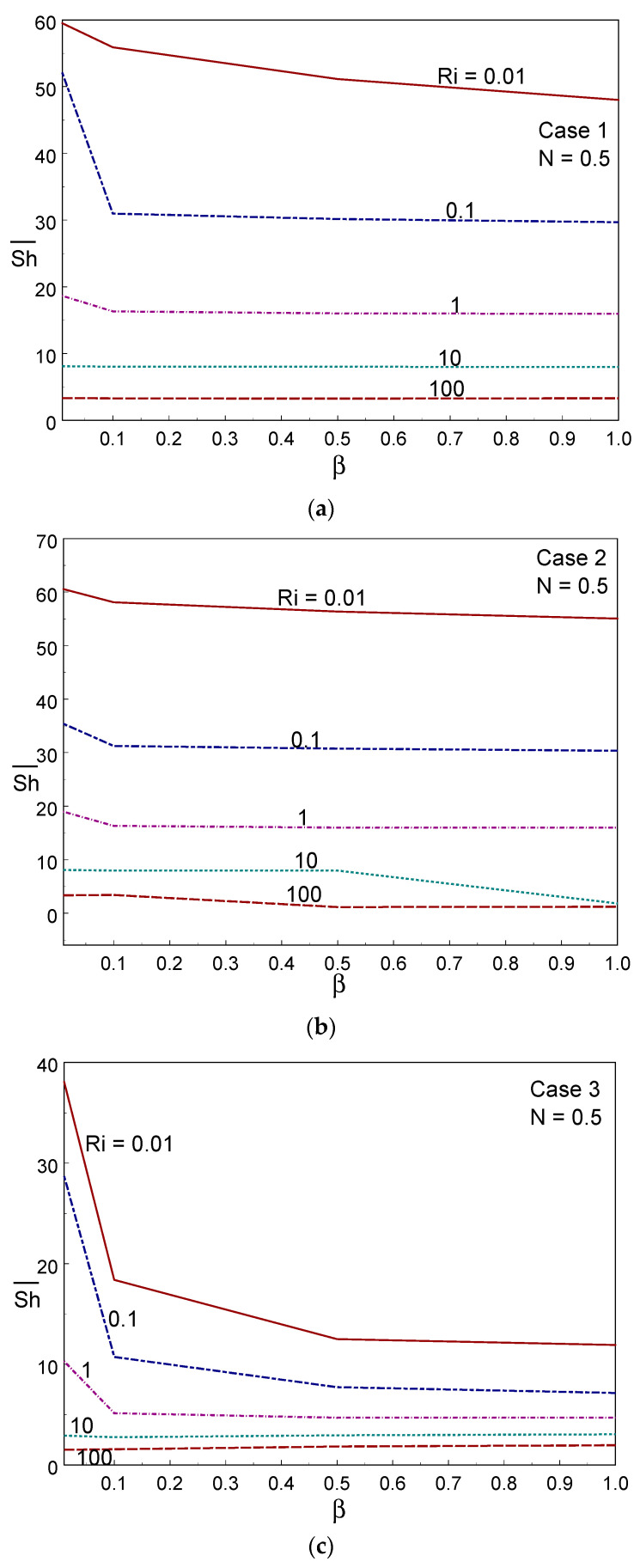
Averaged Sherwood number for different values of Ri and Casson fluid parameters in Case 1 (**a**), Case 2 (**b**), and Case 3 (**c**).

**Figure 13:**
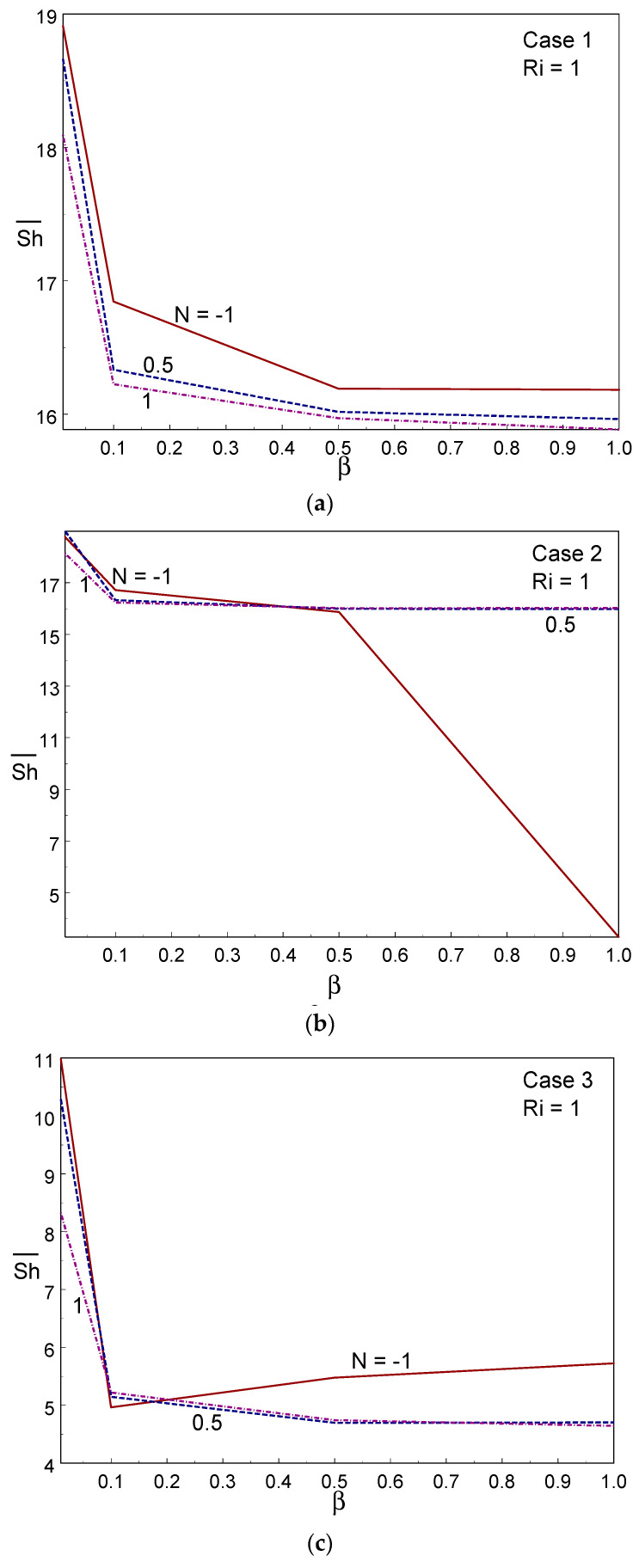
Averaged Sherwood number for different values of N and Casson fluid parameters in Case 1 (**a**), Case 2 (**b**), and Case 3 (**c**).

To explore the mass transport rate, the averaged Sherwood number is illustrated against the Casson fluid parameter and Ri in Figure 12 for the three cases of wall movement. The figure clearly shows that the mass transport rate deteriorated when increasing the Ri value in all three cases; that is, the forced convective regime provided a higher mass transport rate in all cases. Comparing the three cases, Case II offered a higher mass transport rate, whereas case III provided a lower mass transfer rate under all modes of heat mass transfer. At Ri = 100, the mass transfer rate remained constant when changing the value of the Casson parameter. It was also observed that the averaged Sherwood number decreased with increasing β. As a dual cell structure is created due to the same-direction movement of both walls, the fluid was not well-mixed, resulting in a deterioration of mass transfer in case III. Figure 13 shows the effect of the averaged Sherwood number for several values of the CF parameter and buoyancy ratio with Ri = 1. A sudden decline in the averaged Sherwood number was observed when altering the value of β from 0.01 to 0.1, after which it dropped gradually with an increase in the value of the CF parameter. The mass transport rate deteriorated as the buoyancy ratio (N) value increased in case I. However, cases II and III showed the opposite trend; for example, the mass transfer rate was enhanced under case I, whereas a lower rate of transfer was observed under case III. This observation is due to the direction(s) of the moving wall(s). 

**Figure 14:**
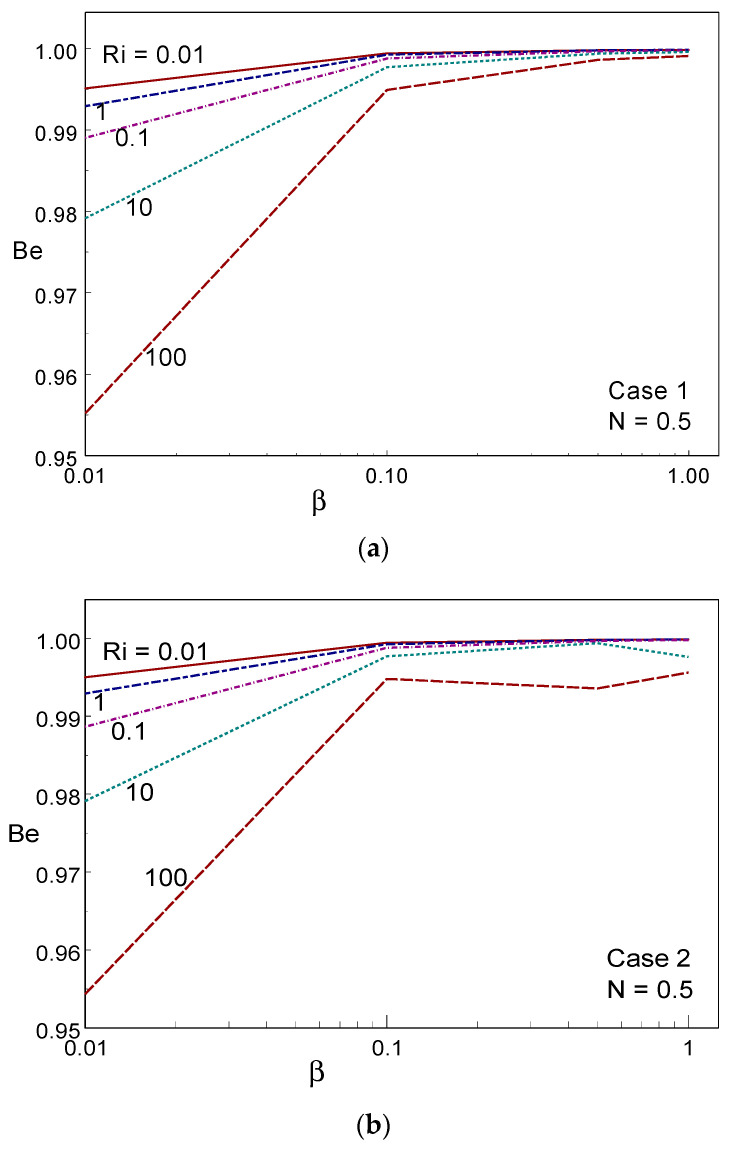

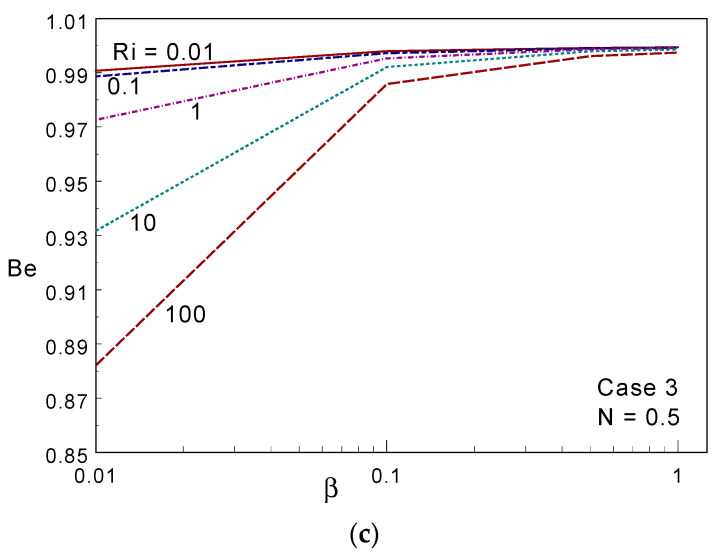
Bejan number for different values of Ri and Casson fluid parameters in Case 1 (**a**), Case 2 (**b**), and Case 3 (**c**).

**Figure 15:**
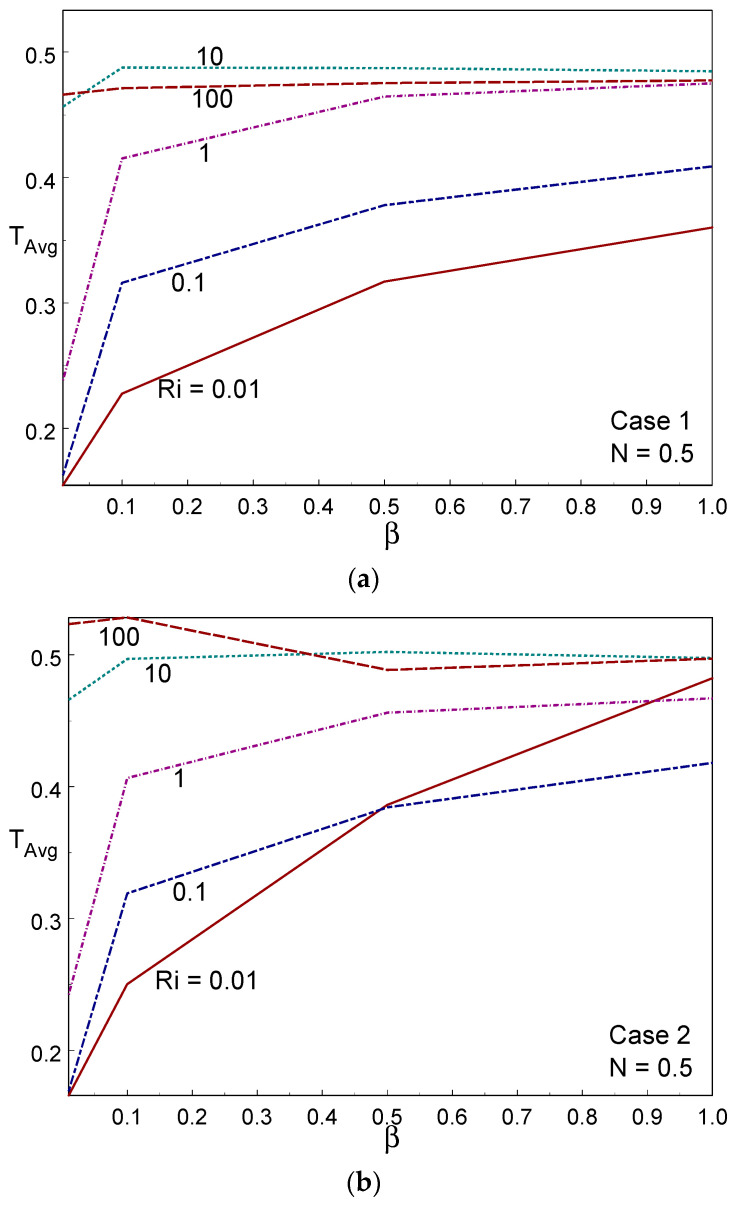

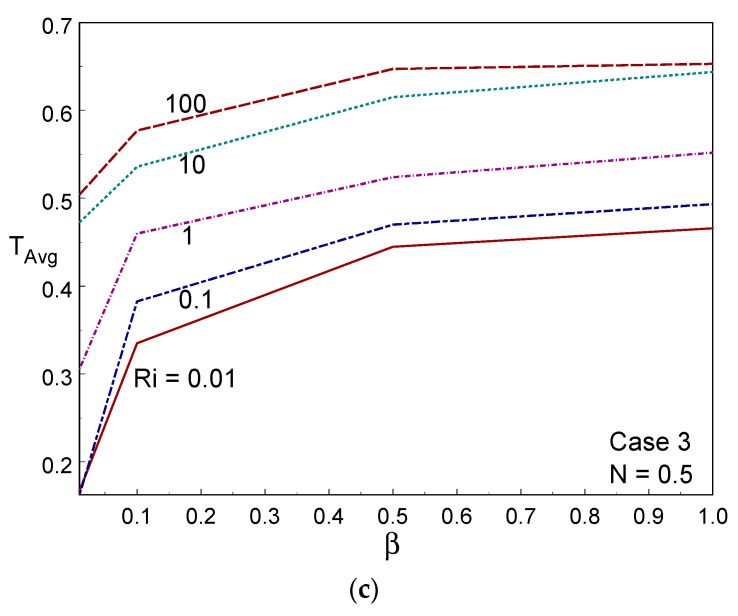
Temp avg for different values of Ri and Casson fluid parameters in Case 1 (**a**), Case 2 (**b**), and Case 3 (**c**).

**Figure 16:**
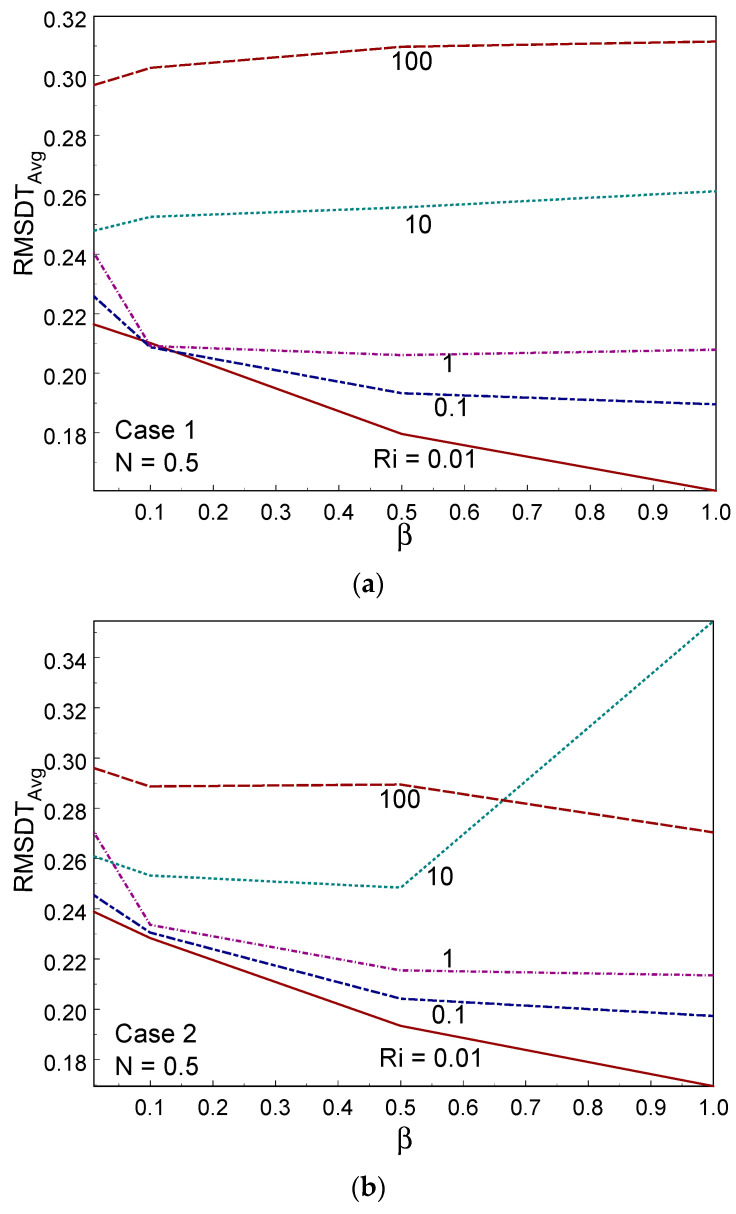

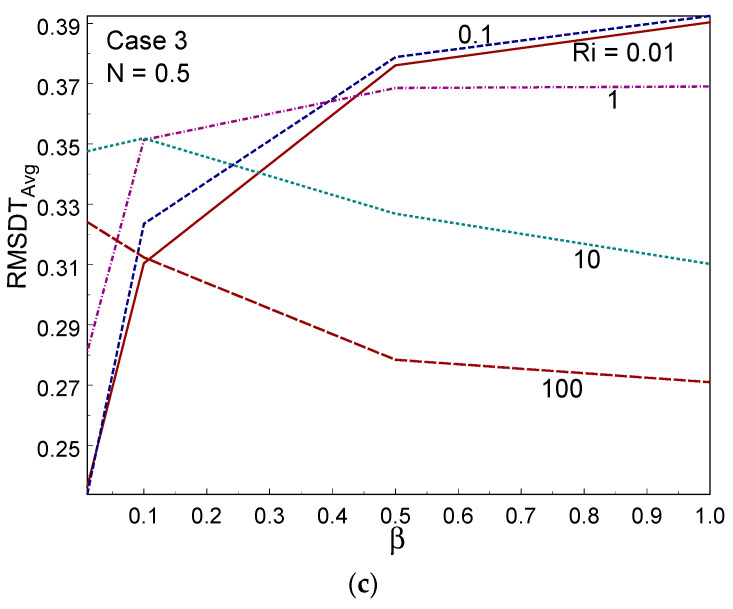
RMSD-Tavg for different values of Ri and Casson fluid parameters in Case 1 (**a**), Case 2 (**b**), and Case 3 (**c**).

Figure 14 displays the Bejan number for numerous values of β and Ri, under the three cases of wall movement. The Bejan number was higher than 0.85 (i.e., Be > ½), indicating that convection heat transmission was dominant in the system. When β = 1, the Bejan number tended to unity (Be→1) for all values of Ri in the three cases. It is evident that heat transport irreversibility is dominant. For lower values of β (β = 0.01, 0.1), the Bejan number increased when decreasing the value of Ri. In all the cases of wall movement, a uniform trend of the Bejan number was observed when increasing the value of β. Figure 15 shows the averaged temperature for several values of Ri, β, and the three cases of wall movement. It can be seen, from these figures, that T_Avg_ increased strongly with increasing Ri and β values for all three cases. The averaged temperature was high (minimum) in the free (forced) convective regime for all three cases. Figure 16 demonstrates the (RMSD-T_Avg_) RMSD averaged temperature inside the container for numerous values of β, Ri, and the three cases of wall movement. The value of RMSD-T_Avg_ increased when increasing the value of Ri in cases I and II; however, it behaved oppositely to Ri in case III. The value of RMSD-T_Avg_ was always below ½.

## 6. Conclusions

The impacts of the movement direction of the wall(s) and entropy generation on doubly diffusive mixed convective stream, as well as the heat and mass transfer of a Casson liquid in a closed container, were numerically explored in this study, for which three cases of wall movement were considered. The following main observations were detected in this study:

➢ Single- or multi-cellular flows were observed, according to the different directions of the moving wall(s). A single stream pattern was seen in case I, whereas a dual cellular stream existed in case III for all values of the considered parameters.
➢ Kinetic energy varied non-linearly with the CF parameter under all convective regimes.
➢ Comparing the three cases, the averaged heat transport was low in case III due to the movement of an isothermal (left) wall against the buoyant force.
➢ The resistance of various liquid layers to their relative motion increased with an increase in the CF parameter, resulting in a decline in the averaged heat transport when increasing the CF parameter.
➢ While strengthening the buoyancy ratio did not support the mass transfer rate in case I, cases II and III presented the opposite trend.
➢ The mass transfer rate was enhanced in case I and diminished in case III due to the direction of the moving walls.
➢ The averaged heat transfer was high under the forced convection mode in all three cases, and was minimal under the free convection mode in all three cases.
➢ The Bejan number increased when increasing the value of the Casson fluid parameter.

## Figures and Tables

**Table 1:** Numerical comparison of $\bar{Nu}$ for a cavity with Rd=0, Pr=0.71, β=∞.

	$\bar{Nu}$	
Ra	Ho et al. [50]	Present
10^3^	1.118	1.103
10^4^	2.246	2.292
10^5^	4.522	4.628
10^6^	8.825	8.935

## Data Availability

Data are unavailable for privacy reasons.
